# Effect of Functional Fillers on Tribological Characteristics of Acrylonitrile Butadiene Rubber after High-Pressure Hydrogen Exposures

**DOI:** 10.3390/polym14050861

**Published:** 2022-02-22

**Authors:** Byeong-Lyul Choi, Jae Kap Jung, Un Bong Baek, Byoung-Ho Choi

**Affiliations:** 1 School of Mechanical Engineering, Korea University, Seoul 02841, Korea; qudfbf1801@korea.ac.kr; 2 Korea Research Institute of Standards and Science, Daejeon 34113, Korea; jkjung@gw.kriss.re.kr (J.K.J.); ubbaek@kriss.re.kr (U.B.B.)

**Keywords:** hydrogen, acrylonitrile butadiene rubber (NBR), wear, functional fillers, pin-on-disc test, blister, micro-pore

## Abstract

In a high-pressure hydrogen environment, the sealing rubber material is swelled by hydrogen, and the mechanical and tribological properties are reduced, causing various problems in the sealing performance. The focus of this study was the effect of the filler type and content on the tribological characteristics of rubber after exposure to high-pressure hydrogen. Acrylonitrile butadiene rubber specimens were exposed to high-pressure hydrogen at 96.6 MPa, and the change in the amount of wear with time after exposure was observed. The wear test was performed using a pin-on-disc ball tip to measure the amount of wear before and after hydrogen exposure of the materials under fixed revolutions per minute and normal load. Scanning electron microscopy was used to observe the wear track and cross section of the specimen to examine the changes in the wear mechanism after hydrogen exposure and to analyze the wear mechanism for each filler. The results of this study are expected to contribute to the evaluation of the tribological properties of the sealing materials used in hydrogen environments.

## 1. Introduction

Since the 18th century, fossil fuels, such as coal, oil, and natural gas, have been the main energy sources for the development of automotive technology and transportation. Despite the benefits of fossil fuels for technological development, environmental problems, such as global warming, have arisen. At the same time, there has been growing concern about the energy crisis caused by the depletion of fossil fuels. Hydrogen has emerged as a promising alternative energy source to fossil fuels. Hydrogen fuel cell electric vehicles (FCEVs) are key elements of the hydrogen economy. Compared to internal combustion engine vehicles, FCEVs are eco-friendly vehicles that produce no pollutants, only generating water and heat in fuel cells. In addition, FCEVs have the advantages of a faster charging speed and a longer driving range than conventional electric vehicles. The FCEV contains a type 4 composite pressure vessel for high-pressure hydrogen storage. Unfortunately, high-pressure containers made of metallic materials may have problems owing to the hydrogen embrittlement phenomenon [1,2,3]. For a long period, many studies on the degradation of mechanical properties (e.g., ductility loss, decrease in fracture toughness, fatigue strength, and fatigue crack growth) have been conducted [4,5,6,7].

In hydrogen stations and FCEVs, polymer materials, such as plastic and rubber, are used to seal the space between each component connected from the high-pressure vessel to the fuel cell system. Rubber materials are mainly used in the form of O-rings, which are repeatedly exposed to hydrogen (up to 87.5 MPa) and a wide temperature range of −50 to 85 °C, depending on their location of use. This severe condition can cause mechanical damage (e.g., micro-sized cracks and blisters) in the O-ring via explosive decompression (ED). When the high-pressure gas is rapidly decompressed, ED occurs inside the O-ring, resulting in internal fracturing [8,9]. Few reports exist on the mechanism of initiation of blisters and the fracture caused by ED [10,11,12,13]. The size and number of blisters are affected by not only the pressure of hydrogen, but also the types and content of the filler in the rubber compound. According to previous research, blisters are more likely to occur in rubber containing carbon black than silica [10,14]. The influence of fillers on the tensile properties of hydrogen-exposed rubber showed similar results to those of blister formation [15,16].

As the FCEV industry has grown in recent years, research on the tribological characteristics of polymer materials in a hydrogen environment has become increasingly undertaken. Polymer seals can be rubbed with a metallic counter face in a high-pressure hydrogen environment. The wear of the seal reduces the sealing performance, as the contact force of the seal decreases with the amount of wear loss. Therefore, it is necessary to evaluate the tribological characteristics of polymer materials in a hydrogen environment. A few reports exist on the tribological characteristics of polymer composites in hydrogen environments [17,18,19,20]. These studies contribute to the understanding of the tribological characteristics of polymers under hydrogen gas. For elastomers, in situ tribological experiments under high-pressure hydrogen have been actively conducted in the last few years [21,22,23,24,25]. Experiments on various types of rubber have been conducted; however, only a few studies were conducted on fillers contained in rubber [25]. In this study, we focused on the effect of the filler type and content on the tribological characteristics of acrylonitrile-butadiene rubber (NBR) after exposure to high-pressure hydrogen. NBR is the most commonly used rubber in a wide range of industries due to its good resistance toward hydraulic fluids, grease, and gas [26,27]. In the FCEV industry, various elastomers, such as NBR, ethylene propylene diene monomer (EPDM), and fluoroelastomers (FKM) are considered for use as sealing material that can survive in a high-pressure hydrogen environment. For the experiment, we selected NBR as the first target prior to other elastomer candidates for hydrogen sealing material. Carbon black and silica were used as fillers, and the contents were classified into 20, 40, and 60 phr. Tribological properties, such as the specific wear rate and wear track morphology, were investigated depending on the type and content of the fillers before and after exposure to high-pressure hydrogen. Hydrogen-induced internal damage appeared differently according to the type and content of the fillers.

## 2. Materials and Methods

### 2.1. Test Materials

An acrylonitrile–butadiene rubber (NBR) plate specimen was obtained from the Korean Institute of Footwear & Leather Technology (Busan, Korea). The main component of neat NBR consists of KNB 35 L with an acrylonitrile content of 34 wt% made by Kumho petrochemical group (Seoul, Korea). Non-filled NBR, N330 carbon-black-filled NBR, and silica-filled NBR were prepared for the test. The size of the primary particle of the N330 carbon black was 28–36 nm. Specific grade Zeosil^®^ 175 silica produced by Solvay (Brussel, Belgium) was used, having a specific surface area of 175 m^2^/g. Each filler was added to the NBR at 20, 40, and 60 phr. The components of NBR, including the filler content, are shown in Table 1.

To prepare the NBR composite, a two-stage mixture was employed using two banbury rotos and an inner mixer with two 8-inch open roll mills. In the first step of mixing (masterbatch), the NBR rubber was pounded and reinforced with fillers, such as carbon black and precipitated silica, and processing aids, such as ZnO, and stearic acid were added to the internal mixer (3 L kneaders, Moriyama Co., Tokyo, Japan). The filling factor was fixed at 0.8, and the starting operating temperature of the Kneader was set to 80 °C. The rotor speed was set to 30 rpm. The NBR rubber was added to a 3 L kneader and masticated for 3 min. Then, the reinforcing filler and the processing aids were incorporated for 10 min. In the second step of mixing, curing agents and accelerating agents were added to the master batch composite, using an open roll mill. The mixer was set to a 3 mm nip opening between rolls. The master batch was added to the roller and mixed for 1 min. Sulfur and TBBS were added in to the batch and mixed for 2 min. The mixing time was same for all composites. Vulcanizate sheets of the composites were prepared by compression molding in a hydraulic press at 150 °C [28].

### 2.2. Test Methods

#### 2.2.1. High-Pressure Hydrogen Exposure Test

A high-pressure hydrogen autoclave at the Korea Research Institute of Standard and Science (Daejeon, Korea) was used for hydrogen exposure. For high-pressure hydrogen exposure, the pressure vessel was purged with argon gas at 5 MPa three times and then filled with hydrogen at a rate of approximately 5 MPa/min to a pressure of 96.6 MPa. The specimens were soaked in high-pressure hydrogen for 24 h. After 24 h, the chamber was decompressed at a rate of approximately 5 MPa/s.

#### 2.2.2. Pin-on-Disc Test (ASTM G99)

**Figure 1:**
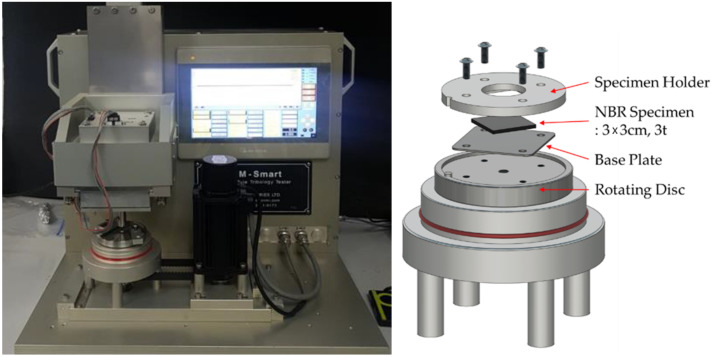
Schematics of the pin-on-disc wear apparatus.

A wear test was performed, using a custom-made pin-on-disc tester (Figure 1) with reference to the ASTM G99 standard. The ASTM G99 standard is based on metallic materials. Therefore, the preparation and testing conditions of the specimens were changed for NBR materials. In this study, through the wear test, the wear loss and morphology of the wear tracks were measured before and after the exposure to high-pressure hydrogen for 24 h. Each test was repeated three times for each test condition. The wear loss was calculated as the difference in the specimen weights before and after the wear test. Before weighing the specimen with an electronic balance, the specimens were stored in a desiccator for more than 24 h. They were then measured. In the case of the specimens exposed to high-pressure hydrogen, the final weight was determined one week after decompression. The weight of the specimen was obtained five times, and the average value was used. We compared the wear characteristics and wear resistance of seven types of NBR with the amount of wear loss, friction force, and coefficient of friction derived from the wear test.

For other mechanical property tests, including tensile and hardness tests, a quick measurement was possible before a large amount of hydrogen escaped from the NBR. However, in the case of the wear test, a certain friction distance or time was required to obtain a sufficient amount of wear loss for a comparison between NBR specimens. At the same time, to evaluate the effect of hydrogen, a wear test had to be conducted while hydrogen was sufficiently present in the specimen.

Depending on the type of filler, even if the same content was included in the specimen, there was a large difference in wear loss. The reinforcing effect of the filler tended to increase as the filler content increased. Nevertheless, the degree of the reinforcing effect depended on the filler type. If the normal load is large, a sufficient amount of wear can be obtained to evaluate the entire specimen type, even in a short-distance wear test. However, in the case of a material with poor wear resistance, the wear may exceed the thickness of the specimen, which may cause problems in the evaluation. Therefore, in this study, 5 N was selected as the final normal load through preliminary experiments.

The rotation speed and total friction distance were the main parameters used to control the testing time. It was necessary to obtain a sufficient amount of wear loss while considering the degassing of hydrogen from the specimen. If a single test requires a long time, only a small amount of hydrogen remains in the specimen. It is thus difficult to determine the effect of hydrogen on the tribological properties. The ball tip used in the wear test was 5 mm from the rotational center. From the preliminary wear tests, the final wear testing conditions were a normal load of 5 N, 300 rpm, and a total sliding distance of 500 m.

To create a uniform contact surface under the specimen, a thin base plate was placed under the specimen and situated on a rotating disc. A customized jig was used to fix the specimen to the rotating disc. When tightening the bolt, a torque of 0.3 kgf·cm was implemented with a torque driver. It was important to use an appropriate torque to prevent bulging at the center, which occurs when the torque implemented at the edge of the specimen is too high. The surfaces of the specimens and ball tips were cleaned with ethanol. After the wear test, the wear particles were collected, and residual small particles on the wear track were removed with a blower. The specimens were stored in a desiccator for at least two weeks until there was no weight change.

#### 2.2.3. Morphology Analysis

**Figure 2:**
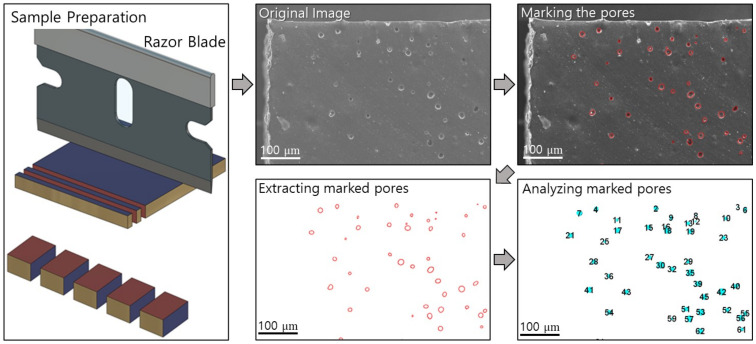
Summary of sample preparation and pore counting procedures after hydrogen exposure via ImageJ.

After high-pressure hydrogen exposure, numerous pores and cracks were created inside the NBR specimens. A scanning electron microscope (JSM-7100F) from JEOL (Tokyo, Japan) was used to observe the wear track and cross section of the NBR specimens. NBR specimens were cut by a razor blade rinsed with isopropyl alcohol. The perimeter of the pores observed in the SEM images was manually marked for pore detection. The centroid, cross-sectional area, and number of pores were counted using free, open-source ImageJ image analysis software. The schematics of morphology analysis were shown in Figure 2.

## 3. Test Results and Discussion

### 3.1. Hydrogen-Induced Internal Damage

#### 3.1.1. Density Change and Blister Formation

**Figure 3:**
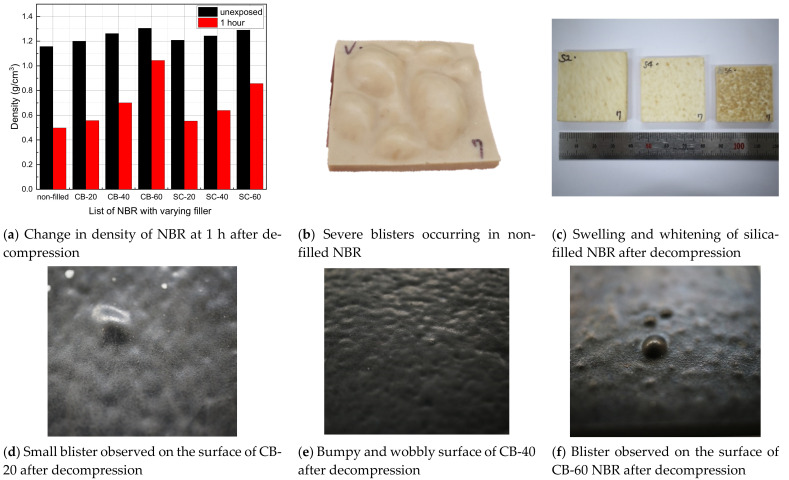
Summary of density changes and blister formation of NBR after hydrogen exposure.

The mass of a specimen before and after hydrogen exposure is measured by employing the electronic balance with the resolution of 10 μg under the environments in which both temperature and humidity are well maintained. The volume before and after hydrogen exposure is measured by the calibrated micrometer under the same environments. Thus, the density is determined by dividing the mass by the volume of specimen. The measured value after hydrogen exposure was taken as the measured one at one hour after decompression started. The density of NBR increased with the filler content, and the density of the carbon-black-filled NBR was slightly higher than that of the silica-filled NBR. After decompression, the NBR began to swell because the dissolved hydrogen molecules aggregate to form small bubbles inside the specimen [29]. Therefore, the density of NBR decreased, depending on the filler content and type (Figure 3a). In some cases of non-filled NBR, severe blisters occurred, as shown in Figure 3b. There was swelling and whitening in the silica-filled NBR, although blisters were not observed on the surface. On the other hand, blisters and surface deformations were observed on the surface of carbon-black-filled NBRs. Because of the small blisters inside the specimen, the flat surface of the unexposed specimen became bumpy and wobbly (Figure 3).

#### 3.1.2. Micro-Pores in Non-Filled NBR

**Figure 4:**
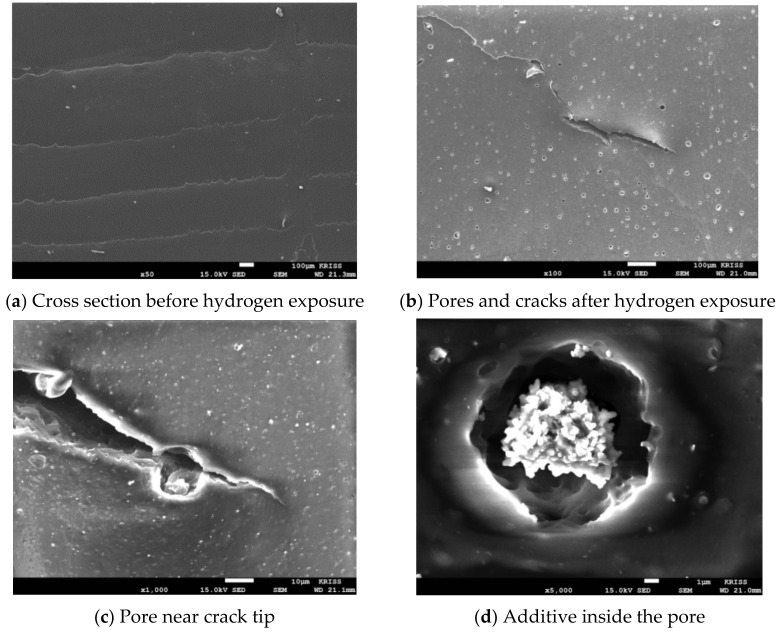
SEM images of non-filled NBR before and after hydrogen exposure.

Blister initiation was categorized for three different cases: blisters formed at a micro-size defect (case 1), blisters formed at the filler agglomerate (case 2), and blisters formed without defects in the rubber matrix (case 3) [13]. In the case of the NBR used in this study, the number and size of bubble formation differed, depending on the type and content of the filler. After decompression, the non-filled NBR had large bubbles on the surface of the specimen. In the cross-sectional area, cracks due to large bubbles were observed. When the additives were aggregated, the gap between the interfaces enlarged, incorporating case 1 and case 2 types of damage. Meanwhile, there was considerable case 3 damage in which no filler agglomerates were observed inside the blister (Figure 4).

**Figure 5:**
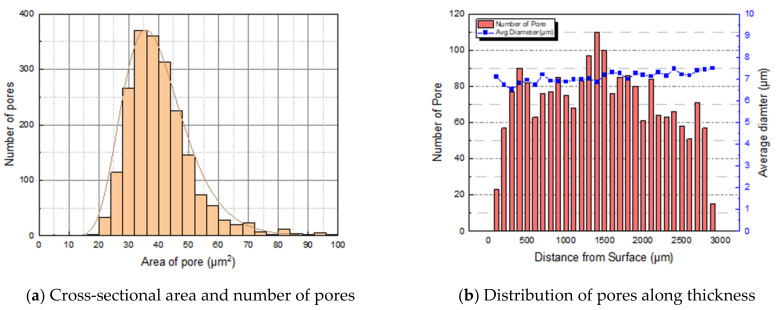
Formation and distribution of pores of non-filled NBR specimen.

To quantify the pores found above, the distribution of pores in the area, including the specimen thickness, was measured. Approximately 2080 pores were found in the cross-sectional area of 6.77 ${\mathrm{mm}}^{2}$. The cross-sectional area between 30 and 40 ${\mu m}^{2}$ included a large portion, as shown in Figure 5a. The pores were counted within a section divided by 100 μm along the thickness of the specimen. The pores were distributed predominantly in the middle of the specimen thickness. As shown in Figure 5b, the average diameter of the pores was calculated from the pores located in the same section. The cross section of NBR containing carbon black and silica was also quantitatively analyzed for hydrogen-induced damage in the same manner.

#### 3.1.3. Micro-pores in Carbon-Black-Filled NBR

**Figure 6:**
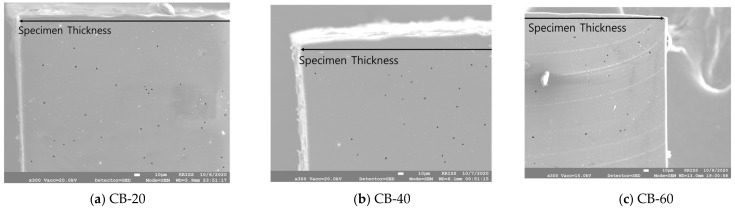
Formation of pores in the cross-sectional area of carbon-black-filled NBR.

**Figure 7:**
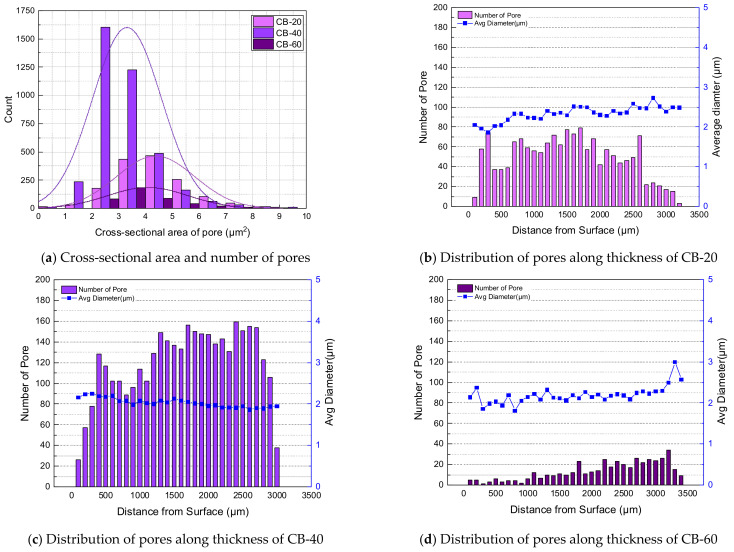
Formation and distribution of pores of carbon-black-filled NBR.

The number, cross-sectional area, and distribution of pores per unit area were measured based on the thickness direction of the carbon-black-filled specimen, as shown in Figure 6. In the CB-filled NBR, pores of similar sizes were formed regardless of the filler content. There were approximately 330 pores per 1 ${\mathrm{mm}}^{2}$ in CB-20, 546 pores in CB-40, and 62 pores in CB-60. The areas of pores per 1 ${\mathrm{mm}}^{2}$ were 0.0014, 0.0018, and 0.0002 mm, and less than 0.2% of the total area was empty as pores. From the CB content of 20 to 40 phr, the number of pores per unit area slightly increased; however, the number decreased significantly at 60 phr. The average diameters of the pores were measured to be 2.33, 2.03 and 2.19 μm, respectively. The carbon black content had a minimal effect on the size of the pores, and the reinforcing effect of carbon black by their content seemed to suppress pore formation. In Figure 7, the distributions of observed pores of carbon-black-filled NBR are summarized.

#### 3.1.4. Micropores in Silica-Filled NBR

**Figure 8:**
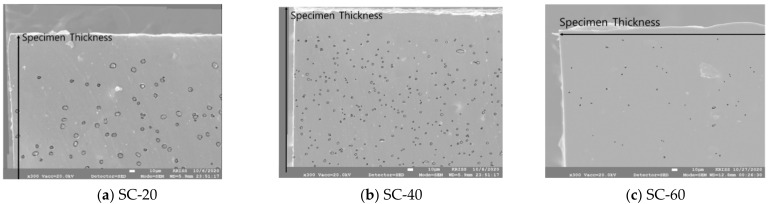
Formation of pores in the cross-sectional area of silica-filled NBR.

**Figure 9:**
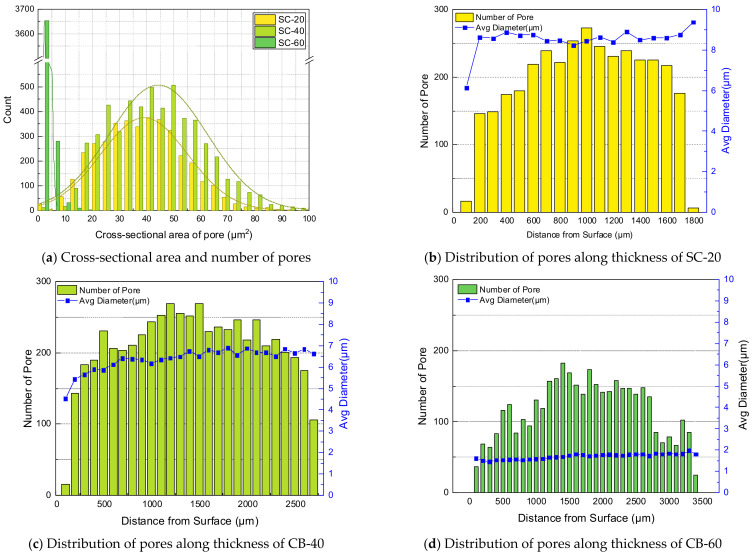
Formation and distribution of pores of silica-filled NBR.

The number, cross-sectional area, and distribution of pores per unit area were measured based on the thickness direction of the silica-filled specimen, as shown in Figure 8. From SC-20 to 60, there were approximately 288, 414, and 745 pores per 1 ${\mathrm{mm}}^{2}$, respectively. The area of pores per 1 ${\mathrm{mm}}^{2}$ was 0.0163 ${\mathrm{mm}}^{2}$ in SC-20, 0.0132 ${\mathrm{mm}}^{2}$ in SC-40, and 0.0017 ${\mathrm{mm}}^{2}$ in SC-60; less than 0.2% of the total area comprised pores. The average diameters of the pores were measured to be 8.5, 6.37 and 1.69 μm, respectively. As the silica content increased, the size of the pores decreased, and the number of pores increased. Silica is considered to be the cause of pore formation; nonetheless, a large amount of silica inhibited the pore size from growing, owing to the reinforcing effect of silica. In Figure 9, the distributions of observed pores of silica-filled NBR are summarized.

### 3.2. Ex-Situ Wear Test

#### 3.2.1. Wear Loss

According to the research on the effect of hydrogen exposure on mechanical properties, mechanical properties such as tensile strength, elongation at break, and hardness were impaired regardless of filler type and content. The relative change in tensile strength at 1 h after hydrogen exposure was inconsistent with seven types of NBR. The relative change in hardness showed the lowest value at 40 phr, regardless of filler type. The results of mechanical property change indicate that mechanical properties of NBR deteriorate after exposure to high-pressure hydrogen. However, these results are not suitable for evaluating the effect of high-pressure hydrogen due to the inconsistency of the relative change in mechanical properties [30]. Therefore, as the specific wear rate and pore density showed distinguishable change after the specimen was exposed to high-pressure hydrogen, it could serve as alternative criteria for the evaluation of hydrogen effects on NBR.

**Figure 10:**
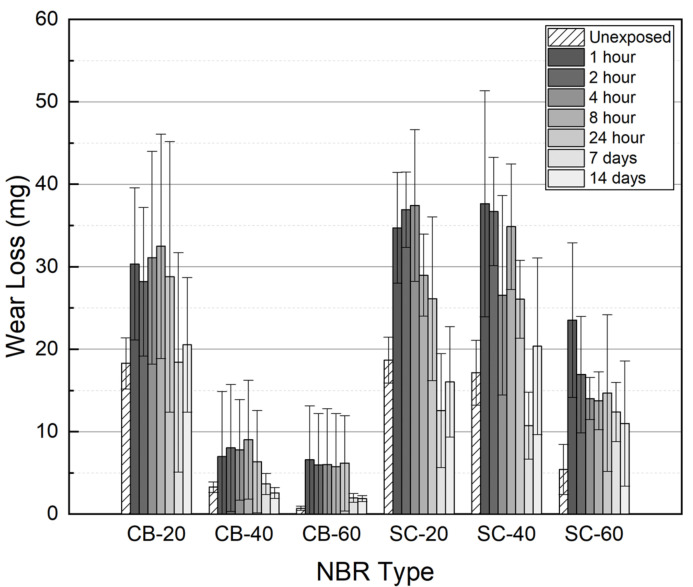
The amount of wear loss measured by elapsed time after decompression.

After decompression, a wear test was conducted on seven types of NBR. The NBR specimens containing filler were tested at a load of 5 N; however, for the non-filled NBR, a wear test was performed at a load of 3 N due to poor wear resistance. Compared to the unexposed specimen, the wear loss of the exposed non-filled NBR increased from 234 to 245 mg. Carbon black showed better reinforcement than silica with the same content. It also seemed that wear resistance roughly recovered over time, although some of the SC-filled silica did not recover to unexposed wear loss, even after 14 days (Figure 10). This can be interpreted as hydrogen exposure causing a permanent change in the tribological properties of the material.

**Figure 11:**
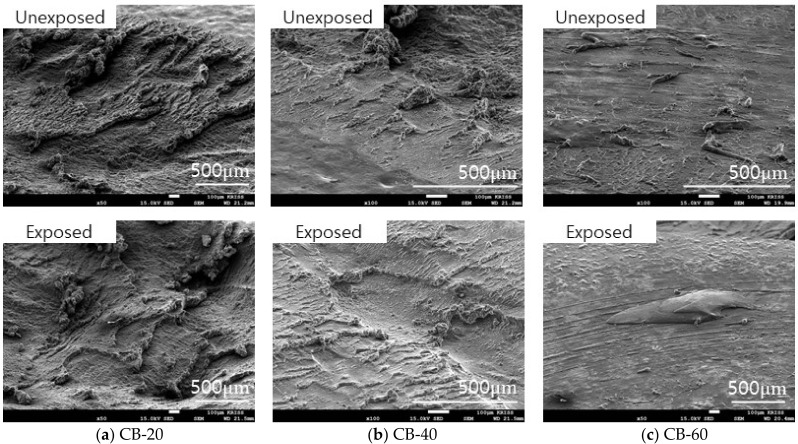
Morphology of the wear track of carbon-black-filled NBR before and after hydrogen exposure.

**Figure 12:**
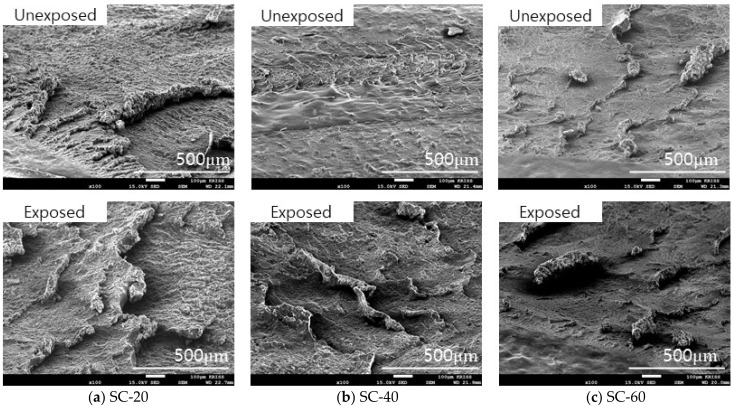
Morphology of the wear track of silica-filled NBR before and after hydrogen exposure.

In the case of CB-20 and CB-40, roll formation occurred on the surface. However, in the wear track of CB-60, roll formation of the material did not occur easily, and the wear particles were fused to the surface as shown in Figure 11. The silica-filled NBR also exhibited a similar wear mechanism on the surface. As the silica content increased, the size and length of the roll formation decreased as shown in Figure 12. The filler content of more than 60 phr made the material harder and tough so that abrasion did not readily occur, and the wear particles did not effectively aggregate.

#### 3.2.2. Coefficient of Friction

**Figure 13:**
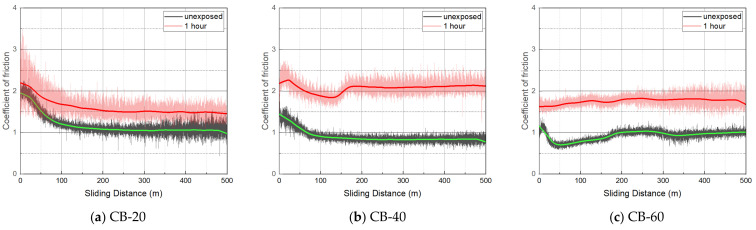

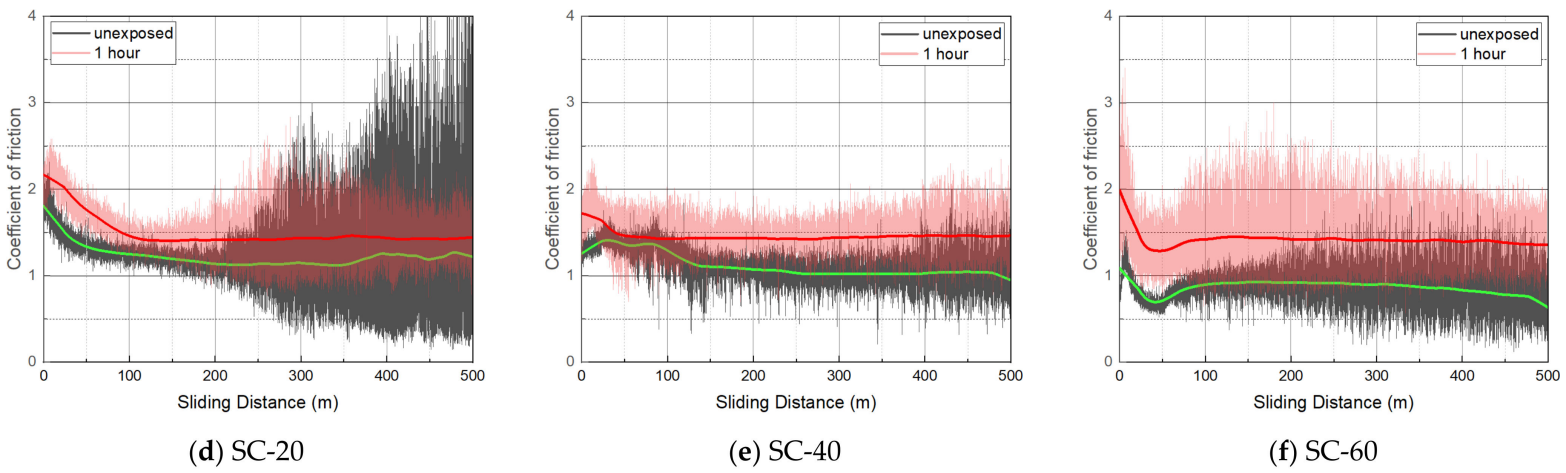
Coefficient of friction before and after hydrogen exposure.

Table 2 shows the average coefficient of friction (CoF) derived from the mean centered line of the plateau region of the CoF curve from a sliding distance of approximately 200–400 m. CoF data between 0 and 100 m were excluded because CoF fluctuated significantly due to the uneven wear track. The CoF of all the specimens slightly increased after hydrogen exposure (Figure 13). The CoF of CB-40 and CB-60 increased by almost 1.5 to 2 times the CoF before hydrogen exposure. The CB-filled NBR showed higher CoF, compared with the SC-filled NBR.

#### 3.2.3. Modified Specific Wear Rate

The wear resistance of the material was compared using the specific wear rates before and after hydrogen exposure. Equation (1) is the specific wear rate, which is the wear volume divided by the product of the applied load and distance. In this study, the wear resistance of each material was compared using Equation (2), which is the wear volume divided by the product of the generated frictional force and the distance. Despite the same wear testing condition, after decompression, the CoF of some materials increased significantly, while showing a low amount of wear loss. Therefore, to include the influence of changing CoF to the equation, the normal load was substituted for the friction force measured from the CoF.

(1) $$W_{s}=\frac{\Delta m}{\rho F_{N}S}$$

(2) $$W_{s}\prime =\frac{\Delta m}{\rho F_{f}S}$$

where Δm is the wear mass loss, ρ is the density of the material, $F_{N}$ is the normal load, $F_{f}$ is the friction force calculated from the mean centered line of the coefficient of friction, and S is the total sliding distance.

**Figure 14:**
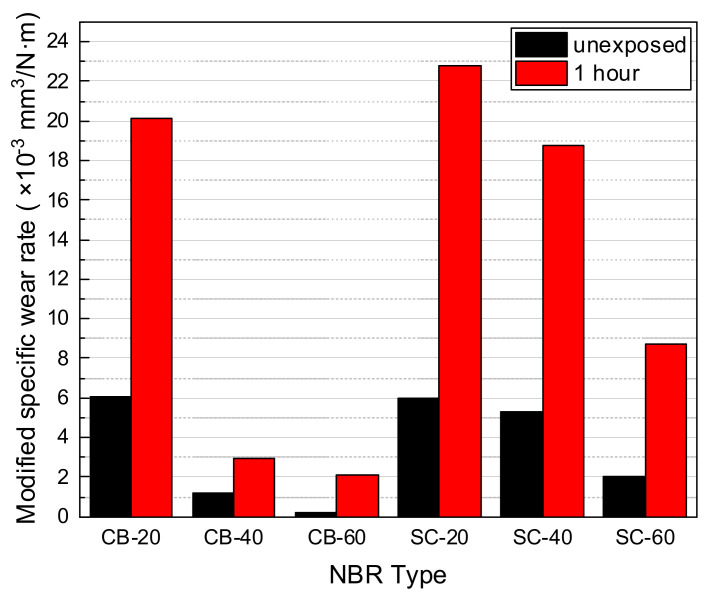
Modified specific wear rate of carbon-black-filled and silica-filled NBR with/without hydrogen exposures.

In Figure 14, the wear test results of carbon-black-filled and silica-filled NBRs for unexposed materials and hydrogen exposed materials with 1 h after decompression are shown. Based on the modified specific wear rate, the wear resistance of the unexposed NBR was ranked as follows: CB-60 > CB-40 > SC-60 > SC-40 > SC-20 > CB-20. After decompression, rank of wear resistance changed into CB-60 > CB-40 > SC-60 > SC-40 > CB-20 > SC-20.

The coefficient of friction curves after decompression became broader and shifted upward. In the wear mechanism of NBR, microcracks are generated and torn apart by deformation at the surface [31]. As the exposed specimen was swollen and soft, larger deformation and wear debris were formed in the wear track. Therefore, the CoF curve fluctuated significantly because of the uneven surface conditions of the wear track.

**Figure 15:**
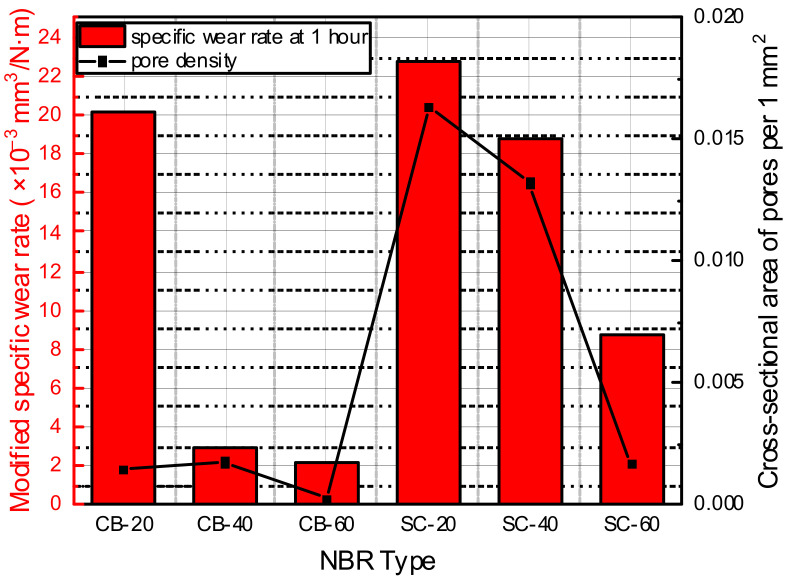
Modified specific wear rate, compared with occupied area of pores per unit area.

According to previous research on the relationship between the hydrogen content and types of fillers, carbon-black-filled elastomers showed higher hydrogen content than silica-filled elastomers [14]. In this study, blisters exposed on the surface were not observed well in the silica-filled NBR; however, numerous micro-sized pores were also formed inside the silica-filled NBR. When the modified specific wear rate after hydrogen exposure and the cross-sectional area occupied by micropores per unit area were compared, similar trends were observed for all materials, except CB-20 (Figure 15). Therefore, it was apparent that micro-sized pore damage inside the material affected the wear properties of the material after hydrogen exposure.

## 4. Conclusions

In this study, an ex-situ wear test was conducted to investigate the change in wear characteristics before and after hydrogen exposure according to the type and content of the filler in NBR. Carbon black and silica were used as fillers, and the specimens were compounded with the filler contents of 20, 40, and 60 phr. The results are summarized as follows:

- The wear resistance before hydrogen exposure tended to increase in proportion to the filler content. In addition, as the size of the filler decreased, the reinforcing effect increased, and thus, the wear resistance increased along with the hardness. Carbon black showed a better reinforcing effect than silica, and similar results were also shown in the ex-situ wear test after hydrogen exposure.
- The specific wear rate and pore density can be evaluating criteria to check the effect of hydrogen exposure on elastomers. Although relative changes of the tensile strength and hardness after hydrogen exposure were small, the specific wear rate and pore density showed distinguishable and permanent changes after hydrogen exposure.
- All specimens showed a similar wear mechanism, that is, roll formation on the wear track, except for CB-60. The wear particles of CB-60 did not separate from the surface of the wear track but adhered to the wear track.
- After high-pressure hydrogen exposure, there were pores and cracks inside the specimen, owing to the high rate of decompression. The number and size of pores changed, owing to the content and type of filler. The cross-sectional area occupied by pores followed a trend similar to the modified specific wear rate. Therefore, it was found that the hydrogen-induced damage was related to the tribological properties of filled NBR.

## Figures and Tables

**Table 1:** Chemical composition of NBR compounds. (Unit: part per hundred resin (phr).)

Materials	Non-Filled	CB-20	CB-40	CB-60	SC-20	SC-40	SC-60
KBR 35 L	100	100	100	100	100	100	100
ZnO	3.0	3.0	3.0	3.0	3.0	3.0	3.0
St/A ^(^^1)^	1.0	1.0	1.0	1.0	1.0	1.0	1.0
N330		20	40	60			
Silica					20	40	60
Si-69					1.6	3.2	4.8
PEG ^(^^2)^					0.8	1.6	2.4
S	1.5	1.5	1.5	1.5	1.5	1.5	1.5
TBBS ^(^^3)^	0.7	0.7	0.7	0.7	0.7	0.7	0.7

^(1)^ St/A: Stearic acid. ^(2)^ PEG: Polyethylene glycol; ^(3)^ TBBS: N-tert-butyl-2-benzothiazole sulfonamide.

**Table 2:** Averaged coefficient of friction after decompression.

Coefficient of Friction	CB-20	CB-40	CB-60	SC-20	SC-40	SC-60
Before hydrogen exposure	1.191	1.856	1.530	1.112	1.032	0.892
1 h after decompression	1.060	0.834	0.985	1.150	1.122	1.089

## Data Availability

The data presented in this study are available upon request from the corresponding author.
